# The role of alcohol consumption in the lives of older Australian women: qualitative insights and an agenda for further research, policy and practice

**DOI:** 10.1186/s12889-024-20083-x

**Published:** 2024-10-05

**Authors:** Simone McCarthy, Hannah Pitt, Kelli Benjamin, Julia Stafford, Danica Keric, Grace Arnot, Samantha Thomas

**Affiliations:** 1 Faculty of Health, Institute for Health Transformation, Deakin University, Geelong, Australia; 2 Cancer Council Western Australia, Perth, Australia

**Keywords:** Alcohol, Women, Older adults, Commercial determinants of health, Risk perceptions

## Abstract

**Background:**

Alcohol consumption presents a threat to the health and wellbeing of women. The alcohol industry often pushes back at global efforts to prioritise the prevention of alcohol harms to women. Qualitative researchers have investigated how younger and midlife women conceptualise their alcohol consumption, but there is very limited research relating to older women (those 60 years and over).

**Methods:**

Using data collected from an online qualitative survey, this paper explored the factors that influence how older Australian women drinkers (*n* = 144. 60–88 years) conceptualised the role of alcohol in their lives. The study used a ‘Big Q’ reflexive approach to thematic analysis, drawing upon sociological theories of risk and symbolic interactionism to construct four themes from the data.

**Results:**

First, alcohol consumption was viewed by participants as an accepted and normalised social activity, that was part of Australian culture. Second, alcohol played a role for some participants as a way to cope with life changes (such as retirement), as well as managing stressful or challenging life circumstances (such as loneliness). Third, alcohol was part of the routines and rituals of everyday life for some women. For example, women discussed the consumption of wine with their evening meal as an important part of the structure of their day. Fourth, participants had clear personal expectancies about what it meant to be a ‘responsible drinker’. They had clear narratives about personal control and moral obligation, which in some cases created a reduced perception of their own risk of alcohol-caused harm.

**Conclusions:**

This research provides a starting point for future public health research examining the factors that may shape older women’s alcohol consumption beliefs and practices. Public health activities should consider the unique needs and potential vulnerabilities of older women drinkers, and how these may be potentially exploited by the alcohol industry.

## Introduction

### The impact of alcohol consumption on the wellbeing of women

There is irrefutable evidence that alcohol consumption presents a particular threat to the health and wellbeing of women [1, 2]. Researchers have documented that the harmful effects of alcohol consumption occur more rapidly and severely for women than men [2], and that some subgroups of women (including women in middle to older adulthood) are drinking at levels that may pose significant risks to their health and wellbeing [3]. There are a number of alcohol-related risk factors that are specific to women [4], including a larger risk of all-cause mortality (as compared to men) for women who drink more than 25g of alcohol per day [5]; breast cancer [6–8]; and the risks to mother and baby associated with alcohol consumption during pregnancy [9]. There are also a range of indirect health and social harms experienced by women from the alcohol consumption of others (including experiences of family related violence) [10].

### The targeting of women by the alcohol industry

While the World Health Organization has stated that no level of alcohol consumption is safe for health [11], the alcohol industry often pushes back at global efforts to prioritise the prevention of alcohol harms to women [12]. This has included using strategies to shape prosocial norms relating to alcohol consumption for women and dispute independent science about harms to women [13, 14]. For example, the alcohol industry has misrepresented the risks of breast cancer from alcohol consumption [13] and has opposed comprehensive public health measures that would provide women with information about the risks associated with alcohol consumption [15, 16].

Instead, the alcohol industry has targeted women for profit [17, 18]. Public relations strategies portray the industry as an active supporter of women’s health and equity through a range of Corporate Social Responsibility (CSR) activities, including sponsoring International Women’s Day [19], and associating their brands with breast cancer charities [20]. Scholars have called for research that moves beyond individual determinants of alcohol consumption in women. This includes investigating the range of social, environmental, and commercial (industry) factors that may contribute to women’s alcohol attitudes and experiences, risk/benefit perceptions and consumption behaviours - and how these may differ across population subgroups, as well as  the range of public health and policy strategies that could be used to respond [10, 21, 22].

### A focus in the qualitative literature on the alcohol consumption of younger/midlife women

To date, much of the focus in the qualitative literature on women’s alcohol attitudes and consumption patterns has been on younger [23–25], and middle aged [26, 27] women. Researchers have documented a diverse range of social, environmental, and experiential circumstances that may influence women’s alcohol attitudes and practices [27,28]. For example, studies show that alcohol is viewed by women as a product that is linked to social connection and group identity [25], is used to construct social identities [29], and helps to relieve life stressors [30, 31], including managing different life stages [32, 33]. More recently, researchers have investigated the range of strategies and tactics that may be used by the alcohol industry to stimulate product consumption in younger and middle aged women and shape positive attitudes towards particular alcohol products [34, 35]. This includes the development of novel products and promotions which seek to capitalise on women’s health fears [30, 36, 37], and which promote alcohol consumption as a mechanism for female empowerment and social connection [21].

What is less clear from current qualitative research is how older women drinkers (60 years and over) conceptualise the role of alcohol and particular alcohol products in their daily lives. For the purpose of this paper, we define older women as 60 years and older. This is based on national reporting statistics in Australia, including the Australian Institute for Health and Welfare National Drug Strategy Household Survey which investigates Australian adults’ use of alcohol, tobacco, e-cigarettes and other drugs [38]. The 2022/2023 survey found that 22% of women aged 60–69 years, and 15% of women over 70 years exceeded the alcohol guidelines by consuming more than 10 standard drinks a week [38]. Barbor and colleagues ([39] p. 36) state that alcohol consumption in older adults is of particular concern because of older adults’ *“elevated susceptibility to harms from alcohol”*, with the factors associated with older age (such as biological changes, health challenges, and the use of prescription medicines) amplifying the harmful effects of alcohol consumption.

### Alcohol consumption, industry tactics and older women

There is very limited research about the alcohol attitudes and consumption practices of older women [40]. This is somewhat surprising given that research indicates that older women consume alcohol more regularly at high-risk levels as compared to younger women [22], with an increasing percentage of older women aged 60 + exceeding the low-risk drinking guidelines [38]. Researchers have found a rise in high-risk drinking among women, particularly in older cohorts, with social, economic, and cultural contexts significantly impacting their drinking behaviours [41, 42]. For example, Kersey and colleagues [41] identified that older women in lower socio-economic areas faced greater alcohol availability and targeted marketing, leading to higher consumption rates. Additionally, older women of higher socio-economic status viewed alcohol as a legitimate part of their social lives, while those with fewer resources were found to use it as a coping mechanism for stress and anxiety [41].

Despite the above, older women (and older adults in general), appear to be largely overlooked in current public health responses to alcohol product harms [40, 43]. There is also limited research that examines the assumption that the alcohol industry will only prioritise marketing towards younger consumers [44]. For example, while researchers have demonstrated that the alcohol industry uses a range of novel strategies to target women, we would argue that there is currently an assumption that younger women will be the main female target audience of the alcohol industry [34]. Older women may also be at increased risk of alcohol industry framings which minimise the harms associated with alcohol consumption, and frame alcohol risks as mostly associated with ‘irresponsible’ patterns of drinking [45]. For example, research by Dare and colleagues [40] showed that while older women stressed the importance of being responsible or staying in control with their drinking, they rarely took into consideration the amount and frequency of their alcohol consumption. This is an important point given that some health risks and harms may result from cumulative alcohol consumption (not necessarily heavy episodic drinking and acute accident or injury).

While a range of population based public health initiatives have been developed to warn people of the potential health and social risks associated with alcohol products, there are very few independent initiatives which have been specifically developed for older adults [46]. Concerningly, alcohol industry funded organisations have developed a range of community interventions which claim to help older adults (over 50 years old) to *“build resilience”* to alcohol problems and make *“healthier choices”* about their alcohol consumption ([47] p. 34). These types of programs may further entrench personal responsibility framings of alcohol risks and deflect from the alcohol industry’s role in alcohol related harm [48, 49].

### Socially constructed alcohol expectancies and boundaries for women

Engagement in activities or products that can be risky or harmful to health may be influenced by a range of socio-cultural contexts, including how individuals situate and prioritise the risks within the contexts of their everyday lives [50]. Risk perceptions and behaviours are also socially constructed and calculated and may be influenced by social contexts and norms, as well as how risks (and benefits) are conceptualised within everyday habits and routines [50]. Zinn [51] describes that risk-taking behaviours can be motivated by a range of factors, including perceived level of control, identity development, and cultural and social contexts, even when individuals are knowledgeable about the risks they are taking. For example, research that has investigated how women conceptualise the risks and benefits of engaging in products that are potentially harmful for their health, such as gambling, found that women downplayed the risks associated with gambling to maintain social identities, engaging in a negotiation in which the perceived social benefits of gambling outweighed the potential and real harms that they experienced [52]. Linked with this are the symbols and meanings that individuals ascribe to the consumption of products, and the impact and influences of socio-cultural contexts and commercial environments in shaping attitudes towards products [53]. This includes the culturally acceptable boundaries and moral obligations associated with the consumption of particular products, and how these may differ between population subgroups. In relation to alcohol, Frank and colleagues ([54] p. 2) argue that while women are increasingly expected and encouraged to be active participants in drinking, they are still *“subjected to traditional discourses of femininity with great implications for how they are assessed on the basis of their alcohol use and engagement in alcohol related practices and identity performances”.* To date, there has been limited research that seeks to understand this from the perspective of older women.

### Study aims and research questions

This study sought to provide a starting point for exploring how older Australian women drinkers (aged 60 years and over) describe the role of alcohol in their lives. The study aimed to provide qualitative insights to inform the development of future public health responses to alcohol consumption in older women. To guide the analysis of data the following three research questions were developed:

- RQ1: How do older women drinkers conceptualise the role that drinking alcohol plays in their lives?
- RQ2: What factors influence their regular and routine engagement with alcohol products?
- RQ3: How do older women frame their perceived responsibilities associated with alcohol consumption, particularly related to times when they would drink more or less alcohol than they normally would?

## Methods

### Approach

The data presented in this paper were part of a larger dataset obtained from an online qualitatively led survey exploring the alcohol attitudes, practices and behaviours of *n* = 500 Australian women (aged 18 years and over) who had consumed alcohol in the last year. The broader survey had a primary focus on alcohol marketing and public relations activities and women’s opinions about policy responses to prevent harms associated with alcohol specifically for women. One other paper has been published from this dataset which investigated women’s attitudes and symbolic consumption practices related to ‘better for you’ alcohol products [30]. Low risk ethical approval was received from the [anonymised].

Qualitatively led surveys provide a different type of data as compared to, for example, in-depth interview studies. Braun and colleagues ([55] p. 641) argue that they are an *“exciting, flexible method”* that are able to provide rich insights into a range of health and social phenomenon. These types of surveys have been successfully applied with children, young people, and adults, across a range of contemporary health and social phenomenon [56–58]. Thomas and colleagues [59] provide a broad overview of the use of this type of qualitative survey in public health research, stating that these types of studies are useful in investigating new public health threats and emerging areas of research focus, and provide important information for agenda setting and policy attention.

There are a range of benefits associated with data collection through these types of surveys, including that they are a convenient way for individuals to participate in qualitative research, they are easily accessible, require minimal time commitment, are able to be completed at a time that suits the participant, and are anonymous (which may enable participants to engage in providing opinions without any fear of judgement) [55]. Further benefits for participants include a degree of autonomy over participation, that participants can take time to reflect on responses, and that participants can drop out of the study easily if the survey does not interest them or meet their expectations [59]. There are also a range of benefits for knowledge users, including a "*wide angle picture*" ([60], p. 70) on issues from a diverse range of individuals. However, as Thomas and colleagues [59] highlight, there are some challenges with online surveys, including not being able to engage individuals in a ‘traditional’ conversation about issues, an inability to explain and clarify concepts, and there may be barriers with written literacy issues. The aim of interpretive qualitative studies is not to provide generalisable data of all individuals in a population and does not seek a single truth about a phenomenon. Rather it aims to provide contextualised understandings of a phenomenon, including understanding the contexts of people’s experiences [61]. We drew upon Braun and Clarke’s [62] Reflexive Thematic Analysis Reporting Guidelines to guide the conceptual and methodological coherence and rigour of the study, including refining specific questions to guide the analysis of the data used in this specific paper.

### Criteria for recruitment

To investigate the specific experiences of older women who consume alcohol, this paper focuses on the responses of the *n* = 144 women aged over 60 years old who participated in the survey. We asked participants which gender they identified with as a screening question, so we assume that those who completed the survey identified as women (although we did not ask any further questions that allowed participants to elaborate on their gender identity).

Women were invited to take part in the survey through Qualtrics, an online survey platform and panel company. The survey was created using Qualtrics, and it was distributed to eligible participants registered with their partner panel companies. Participants who are registered to these panel companies accumulate points for each survey that they complete. Potential participants could download the Plain Language Statement and click to confirm their consent to participate prior to beginning the survey questions. To ensure diversity in the sample, soft quotas were set for geographical region and age.

### Data collection

The broader survey was piloted in September 2022 which enabled the research team to ensure the survey worked from a technical and comprehension standpoint. The survey was then adjusted with minor changes to the wording of some questions, the pilot responses discarded, and the complete survey launched in late September 2022 with data collection continuing until all the data was collected in October 2022. The research team reviewed the pilot survey responses to ensure that all participants had made genuine attempts to complete the survey, and there was no nonsensical or incomplete data. For example, participants who wrote random letters in response to questions, or ‘N/A’, ‘I don’t know’, or ‘not sure’ for the majority of their responses to qualitative questions were removed from the survey.

In relation to the data presented in this paper, a range of discrete questions were asked about socio-demographic status (age, state of residence, level of education, and employment status), and alcohol characteristics (how often they consumed alcohol, which products they purchased and preferred, who they drank alcohol with, and at what locations). To estimate alcohol consumption, women were shown a figure illustrating different products and the equivalent standard drink measure (for example, 375 ml full strength beer = 1.4 standard (std) drinks, 375 ml light beer = 1.0 standard drink, 30 ml spirits = 1.0 std drink and 150 ml wine = 1.6 std drinks). Women were asked how many standard drinks they would usually consume on a day that they had an alcoholic drink. They were then asked to qualitatively explain their main reasons for drinking alcohol, the factors that influenced their alcohol consumption, and the times that they would drink more or less alcohol than they normally would.

After the collection of all of the data, the dataset was prepared for data interpretation [59]. As Thomas and colleagues [59] explain, the quality and type of data that is collected in these types of surveys can vary and decisions need to be made about the participants (or responses) to be included in the dataset and why. We looked to see if participants had made a bone fide attempt to answer all or most of the questions, removing nonsensical responses (including random letters). We also looked to ensure that responses were coherent across questions, and that there were few inconsistencies in responses (for example participants saying they had consumed alcohol in the screening question for the survey, and then saying they did not consume alcohol in other parts of the survey). We had discussions within the team to check that there was broad agreement about the participants that we should remove from the dataset.

### Data interpretation

Quantitative data relating to socio-demographic and alcohol characteristics were analysed in SPSS using descriptive statistics.

Qualitative open text data were interpreted using Braun and Clarke’s [63] six phases of Reflexive Thematic Analysis. We used what Braun and Clarke ([62] p. 3) refer to as a ‘Big Q’ approach, which embraces researcher subjectivity and interpretivist paradigms, views *“knowledge as situated and partial”*, and which use an *“organic and flexible approach to coding that is responsive to the researcher’s deepening engagement with their data, and their reflexivity”*. As public health researchers we take the position that alcohol is not an ordinary commodity, and is a toxic substance that has the potential to cause significant health harms to individuals and communities [39]. We recognise that the risks and harms associated with alcohol are shaped by a range of individual, social, environmental, and commercial (e.g. the practices of the alcohol industry) factors that are situated within the policy environments that regulate and place boundaries around the way in which the alcohol industry can provide and promote its products in different settings.

Data were coded in relation to the three research questions, as well as theoretical assumptions about risk behaviours and environments [50]. For example, we considered Zinn’s key dimensions in risk-taking; motivation, control, reflexivity and identity [51], and symbolic consumption [53] when coding and constructing themes from the data. This included considering the social contexts and circumstances of participants' drinking practices and behaviours, and the broader alcohol environments in which they were situated. For example, we considered the contexts in which women mostly engaged in drinking (such as the home), their perceived social acceptance of drinking, and who they reported drinking with. Collaborative discussions with the research team were then held to explore interesting ideas, challenge preconceptions, and scrutinise initial perspectives. There was collective input in decision-making about the approaches we took in constructing themes, and in ensuring approaches to coding were interpretivist rather than positivist [62]. Codes were grouped together to create thematic clusters. This involved the research team synthesising codes that shared a similar conceptual meaning to form a broader level theme. Themes were refined to ensure coherence and relevance to the research questions and the overarching narrative. Each theme underwent rigorous review to ensure the theme conveyed a compelling story reflective of shared meanings across participant responses. Themes collectively highlighted key patterns within the dataset. Reflection on these themes continued during the write up of the manuscript, with further refining of the themes to ensure clear separation between and clarity of the themes.

## Results

### Socio-demographic and alcohol characteristics

Table 1 provides detail about the socio-demographic and alcohol characteristics of the *n* = 144 older women who participated in the study. Women ranged in age from 60 – 88 years (mean: 68.74; SD: 6.1). Three-quarters of women (*n* = 107, 74.3%) were residents of Australia’s three largest states—New South Wales, Victoria, and Queensland. One in five (*n* = 30, 20.8%) had completed a tertiary level of education, and over half were retired (*n* = 96, 66.7%). In relation to alcohol consumption, about three-quarters said that the place they mostly consumed alcohol was in their own home (*n* = 107, 74.3%), and nearly one-third (*n* = 40, 27.5%) stated that they typically consumed alcohol alone. Over half of the sample said that they had consumed alcohol at least once a week in the last twelve months (*n* = 86, 59.7%), which included one in five (*n* = 30, 20.8%) who had consumed alcohol 5–7 days of the week. Slightly under a quarter of the sample (*n* = 34, 23.6%) reported consuming three or more standard drinks on each drinking occasion, and a tenth of the sample (*n* = 15, 10.4%) consumed more than four standard drinks on a single occasion at least weekly.

**Table 1:** Socio-demographic and alcohol-related characteristics of older Australian women drinkers aged 60–88 years (*n* = 144)

**Socio-demographics**	**Frequency**	**Percentage**
**Geographic location**		
New South Wales (NSW)	47	32.6%
Queensland (QLD)	33	22.9%
Victoria (VIC)	27	18.8%
Western Australia (WA)	18	12.5%
South Australia (SA)	9	6.3%
Tasmania (TAS)	6	4.2%
Australian Capital Territory (ACT)	3	2.1%
Northern Territory (NT)	1	0.7%
**Age**		
60–69	78	54.2%
70–79	60	41.7%
80–89	6	4.2%
**Education**		
Secondary school education (year 10–12)	64	44.4%
Trades-based (TAFE) education	50	34.7%
Tertiary education	30	20.8%
**Employment status**		
Retired	96	66.7%
Working part-time or casually	21	14.6%
Working full-time	11	7.6%
Homemaker	9	6.3%
Unemployed but looking for work	2	1.4%
Other	5	3.5%
**Alcohol characteristics**	**Frequency**	**Percentage**
**Frequency of alcohol intake in the last 12 months**		
Everyday	14	9.7%
5–6 days/week	16	11.1%
3–4 days/week	20	13.9%
1–2 days/week	36	25.0%
2–3 days/month	21	14.6%
Once a month or less	37	25.7%
**Personal estimate of no. of std drinks consumed when drinking**		
½—1 drink	58	40.3%
2 drinks	52	31.9%
3–4 drinks	29	20.1%
5–8 drinks	3	2.1%
7–8 drinks	1	0.7%
9 or more drinks	1	0.7%
**More than 4 std drinks in a day**		
Never	82	56.9%
Once a month or less	44	30.6%
Fortnightly	3	2.1%
Weekly	8	5.6%
2–3 times per week	3	2.1%
4–6 times per week	2	1.4%
Daily	2	1.4%
**Where do you normally drink?**		
In my own home	107	74.3%
At licensed premises (e.g., bars, pubs, nightclubs)	13	9.0%
At a friend/family members house	13	9.0%
At restaurants/cafes	11	7.6%
**Who do you normally drink with?**		
With your household (e.g., partner/housemate)	72	50%
By myself	40	27.8%
With friends	23	16%
With extended family	7	4.9%
Other	2	1.4%

### Qualitative themes

**Fig. 1:**
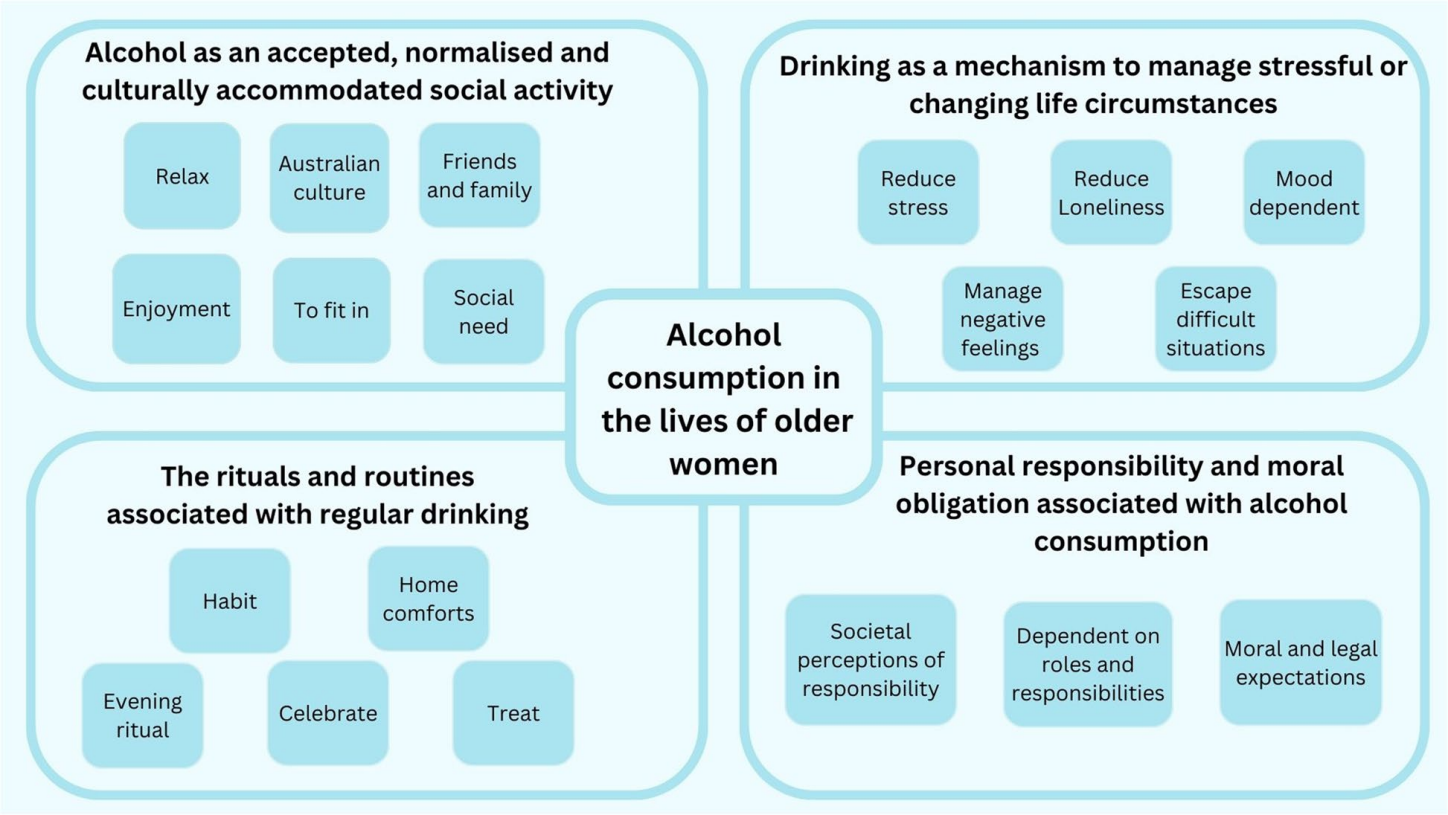
Alcohol consumption in the lives of n=144 older Australian women

Four themes were constructed from the qualitative data: 1) Alcohol consumption as an accepted, normalised and culturally accommodated social activity; 2) Drinking as a mechanism to manage stressful or changing life circumstances; 3) The rituals and routines associated with regular drinking; and 4) Personal responsibility and moral obligations associated with alcohol consumption. Figure 1 provides an overview the role of alcohol consumption in the lives of participants.

#### Alcohol consumption as an accepted, normalised and culturally accommodated social activity

Participants framed alcohol consumption and the role of ‘drinking’ as an accepted and highly normalised social activity. Alcohol had a range of functional and symbolic roles in the daily lives of women. Some women stated that they thought that alcohol played a role in enhancing their lives through its ability to relax them and provide them with a moment of enjoyment. Increased positive emotional and social feelings were a common feature in participant responses, regardless of whether consumption occurred with others or by themselves:

*“I like it and it makes me feel comfortable, something to look forward to at the end of the day”* – 76-year-old, retired, NSW.

Participants often emphasised how drinking had a symbolic meaning as part of the values and norms of Australian culture. For example, one 77-year-old woman stated that drinking alcohol was *“part of the Aussie lifestyle”,* emphasising that drinking went hand in hand with socialising in Australia. Others talked about the ingrained drinking cultures that had been *“the norm”* when socialising since they were young. There was also a clear emphasis on drinking within social networks – even for those women who said they mostly drank at home alone. Friends and family were central to older women’s lives and their drinking experiences commonly revolved around what was happening in these social spheres. For example, this included drinking alcohol when catching up with family, out with friends, entertaining dinner guests, and at social work functions. One 70-year-old woman described her social drinking as central to sharing time with her loved ones:

*“I suppose it relaxes me and it is a nice way to share time with my husband or friends”* – 70-year-old, retired, NSW.

However, while some women indicated that they drank to fulfil a social need, others stated that they were more inclined to drink due to a feeling of social pressure. This included wanting to keep their partner company while they drank, or to join in with others who were drinking. Some described changing their drinking patterns *“to fit in”* or *“to go with the trend”* and emphasised companionship as a prominent influence on their drinking. This led women to increase the amount of alcohol that they purchased or consumed, particularly when hosting social events:

“*I usually will buy more if I entertain friends at home or* [for] *special occasions. Otherwise, my buying habits are normal”* – 65-year-old, retired, Victoria.

#### Drinking as a mechanism to manage stressful or life changing circumstances

For some participants, alcohol consumption was perceived as a method of regaining control over their emotional health, with several women stating that they used alcohol to reduce stress, or wind down at the end of the day. For example, a few women stated that they would drink more alcohol than usual when they were *“really stressed”* or to *“chill out”.* Some women’s responses revealed a degree of social disconnection, using alcohol to help them to manage feelings of low self-confidence and loneliness. One 60-year-old woman who was retired acknowledged that loneliness played a role in her increased alcohol consumption. She stated that she drank more when she *“felt lonely*” and less *“when I’m happy and with my family”.* Others stated that their alcohol consumption was influenced by *“my mood”* and the stressors in their lives. For a few women who drank regularly (at least weekly), alcohol was used as a direct method of coping. For example, some women explained their reason for drinking as an escape, to drown their sorrows, or to forget about difficult situations:

*“Being able to escape all the bad things”* – 64-year-old, retired, Victoria.

Other women cited emotional or physical circumstances that influenced their drinking, stating that they used alcohol to manage feelings of unhappiness, anger, depression, grief, or physical pain:

*“Pain, or to cheer me up or angry or lonely and depressed”* – 68-year-old, retired, NSW.

#### The rituals and routines associated with regular drinking

For many women, the consumption of alcohol was described as being part of their regular daily routine. Some women stated that drinking was a routine that they had developed from when they were younger. These women referred to it as “*a habit*”, which was most prominent in participants who mostly drank at home, as it centred around home comforts. Many women referenced drinking in terms of its symbolic function in signposting the beginning or end of something:

*“It finishes off my day before I cook and have dinner. It relaxes me and puts me in a good place”* – 63-year-old, working part-time, QLD.

There were very specific consumption rituals associated with wine. Wine was symbolically ritualistic and was associated with preparing a meal or having dinner, but also was associated with celebrations and special occasions. For example, wine was an alcohol product that was more embedded into daily life, with other forms of alcohol (such as beer or spirits) rarely mentioned in this context:

*“It gives me a boost and I guess it has become a habit to have a glass of wine with dinner. Association with a cooked meal and so sipping through the meal is a "necessary" habit”*– 81-year-old, retired, NSW.

*“I enjoy a glass of wine with a meal”* – 50-year-old, working full time, Victoria.

For those women who only drank occasionally, wine was the product they identified as being used to celebrate special occasions:

*“An occasional after dinner wine to celebrate something” –* 62-year-old, retired, WA.

*“If I have something to celebrate”* – 60-year-old, working part time, Tasmania.

Despite many women describing alcohol consumption as being embedded in the routine of their daily lives, these women still perceived that drinking was only appropriate in certain contexts. For example, the regular use of phrases like *“I only drink when […]”* epitomised the concept of acceptability in many women’s responses. This included both purchasing and drinking practices that were often expressed as ‘occasional’. Alcohol consumption was also expressed by some women as a treat. These women saw alcohol as a product that they looked forward to and rewarded themselves with:

*“Habit, I look forward to something to treat myself”* - 78-year-old, retired, ACT.

#### Personal responsibility and moral obligations

About a third of older women said that they mainly consumed alcohol when they were on their own. However, even though women in this group regularly consumed alcohol, they were confident they had their drinking ‘under control’. They indicated explicitly that they did not *“over drink”* and that they were aware of what they considered to be ‘normal’ levels of drinking. Older women represented themselves as thoughtful, responsible drinkers. This reflected adherence to well-established cultural and social norms of what they perceived were acceptable or responsible patterns of alcohol consumption. While some women in the study perceived retirement as a time of reduced responsibility, which contributed to an increase in alcohol consumption, a few women described retirement as a time of reduced alcohol consumption because they had fewer stressors:

*“Retirement [*means I*] don't have to worry about as many things as I used to”* – 70-year-old, retired, QLD.

Older women’s responses highlighted changes in drinking practices across various life stages, with societal perceptions of responsibility associated with age influencing these changes. Many women spoke about the drinking cultures they experienced earlier in their lives, often referring to their *“younger days”*, when they enjoyed greater freedom and less responsibility. During this period, they recounted partying and drinking with the intention of getting intoxicated:

*“Many years ago when I was younger partying”* – 78-year-old, retired, NSW.

*“When I was younger used to drink to get drunk”* – 72-year-old, retired, NSW.

Participants were also aware of how their drinking practices might be perceived by others, particularly when drinking outside the home. There was a perception that excessive or over consumption of alcohol was less socially acceptable for older women. They referenced their gendered supporting roles and caregiving responsibilities that shaped their drinking practices and described the need for them to be ‘responsible’ with their drinking when they had been pregnant, were active as a parent or grandparent, or were driving or engaging in work-related responsibilities:

*“If I’m driving, I won’t drink anything at all. Or if responsible for a minor and want to have no alcohol in my system”* – 60-year-old, homemaker, NSW.

For these women, their practices aligned with what they perceived was expected of them in these roles, adhering to both moral and legal standards. For example, one woman stated, *“when I have to drive, I don't drink alcohol”.* A few women discussed engaging in regular drinking but in a way they perceived to be responsible or risk reducing. This included having just the *“one drink occasionally”* and limiting themselves to *“a glass of wine with a meal”.* Even for those women who drank regularly, it was clear that they believed they could alter their drinking practices at any moment.

## Discussion

This study aimed to explore the factors that influence how older Australian women conceptualised the role of alcohol in their lives, the routines and rituals associated with their alcohol consumption, and the perceived responsibilities and moral obligations associated with drinking. These findings raise three points for discussion.

Many of the participants in this study consumed alcohol regularly, and these patterns of consumption were part of the routines and rituals in their daily lives. Contributing to this were factors associated with the normalisation of drinking within social and cultural contexts [40, 64], including drinking because of social pressures or to fit in, which are concepts more commonly investigated with young people. While for some women, drinking remained occasional in social situations, other women maintained regular patterns of consumption – particularly associated with wine. These women used alcohol for social connection, or to manage stressful or changing life circumstances. While there were clear social drivers of alcohol consumption for some women, for others, drinking was an activity that they engaged in alone. Similar to studies with women in midlife, alcohol was viewed as an easily accessible and convenient product that was used as a coping mechanism for stress relief, or to manage difficulties and feelings of vulnerability [33, 64]. What is less clear from the data is the extent and nature of the stressors that older women were experiencing in their daily lives. Investigating how alcohol use may intersect with health, social and economic stress will be important in further understanding the drivers of older women’s alcohol consumption practices.

Alcohol is arguably a product that is normalised as one that can help individuals cope with negative experiences, and may be particularly socially and culturally acceptable if women feel that they are adhering to the responsible drinking narrative promoted by the alcohol industry. This study highlights the ongoing influence of Australia’s deeply ingrained drinking culture on generations of women. While there is evidence that these cultural patterns of drinking, and the social position of drinking may be changing for younger generations in Australia and elsewhere [65], a range of societal constructs and historical traditions support older women’s drinking behaviours [40, 66]. These include how particular alcohol products may be seen as more normalised, acceptable or embedded for particular population subgroups [30]. Qualitative research is able to contribute important theoretical perspectives about how social meanings and practices relating to alcohol are shaped [67] and may provide novel insights into how the alcohol industry may influence drinking practices.

Participants discussed their perceived personal responsibilities and moral obligations associated with alcohol consumption, and in particular the gendered cultural expectancies of alcohol consumption for women. They depicted themselves as making conscious, rational decisions regarding their alcohol intake and described their alcohol consumption as responsible, even when they were drinking regularly. Women’s narratives about responsible alcohol consumption appeared to align with how the alcohol industry portrays responsible drinking. The alcohol industry uses responsible drinking messaging as a strategically ambiguous term that allows for multiple interpretations [68, 69]. ‘Irresponsible consumption’ is presented as binge-drinking, drink-driving, and negative consequences for the individual and those around them [68]. These examples align with the themes constructed from the perceptions of older women drinkers in this study. Many women therefore might not align their alcohol consumption with health guidelines, relying instead on what they view as socially acceptable in their peer groups. Given the subjective nature of how 'responsible drinking' is interpreted and the influence of the alcohol industry’s messaging, it is vital to acknowledge the social contexts in which older women consume alcohol. Public health initiatives risk being ineffective if they fail to recognise and address these social contexts. This finding emphasises the need for nuanced public health interventions tailored to older women which take into consideration how they may conceptualise the risks and potential harms associated with alcohol consumption [70, 71], as well as the impact of alcohol industry’s responsible drinking messaging on alcohol consumption, beliefs, and attitudes. This may include developing messaging that disrupts and seeks to reframe older women’s prevailing notions of ‘responsible drinking’. There is a noticeable gap in public health messaging that explicitly targets older women, helping them see themselves as the intended audience for intervention. When they mostly see messages that normalize regular drinking or promote 'responsible drinking' by the industry, these become their main reference points instead of health information. Effective public health strategies should explicitly address these social dimensions and provide clear, relatable guidance that resonates with older women, helping them make informed decisions about their alcohol consumption.

Finally, the findings of this research highlight the need for alcohol policy approaches to consider gender and age, and for measures to counter the influences of the alcohol industry. These approaches can help reduce potential harms to older women and protect their health. The World Health Organization encourages governments to consider gender when developing their alcohol policies, primarily due to alcohol industry marketing which is increasingly targeting women [72]. The World Health Organization [72] highlights that the industry is increasingly using gendered approaches to appeal to consumers, and that it is vital for public health responses to keep pace with these changing marketing tactics. While current research demonstrates that younger women are targeted by the alcohol industry (through the creation of new products, the use of lifestyle messages that are underpinned by gender stereotypes, offers of feminised accessories, and messages of empowerment [73] we should not assume that the industry are only interested in the younger female market. It is possible the alcohol industry will continue to promote its products to any population subgroups that they think will be profitable. Older women may be an emerging target market for the alcohol industry, and may arguably fly under the radar for public health and health promotion interventions if research interest is solely focused on younger populations. Researchers (and funders) should continue to investigate the influence and effects of gendered alcohol marketing across different population groups, including among older populations. Industry funded groups are also developing community based interventions specifically targeted towards older adults. This may also highlight the need for independent (i.e. not linked with the alcohol industry) public health and health promotion responses which consider the unique needs of older women, including how they may be vulnerable to commercial alcohol marketing tactics. Older women are not a homogenous group, and consideration should also be given to how different groups of older women may conceptualise any risks or perceived benefits of alcohol consumption in their daily lives, and how they may be targeted by alcohol industry marketing, corporate social responsibility, and public relations strategies.

## Limitations

This study relied on survey data obtained from a select group of women drinkers who had chosen to participate in online panels. It relied on participants’ ability to estimate their alcohol intake based on a description of standard drink measures. Any misunderstanding of measures, or inability to calculate the number of standard drinks they consumed, means that personal estimates may not be reliable indicators of consumption.

## Conclusion

This study has provided a starting point for future public health research examining the factors that may shape older women’s alcohol consumption beliefs, practices and behaviours, including their regular engagement with alcohol. Given the insights from the women in this study, we would recommend that further public health research and policy attention is needed in the following areas:

1. The factors that influence how older women (including women who are non-drinkers or former drinkers) conceptualise the risks and harms associated with alcohol consumption.
2. The influence of alcohol industry messaging on older women’s alcohol knowledge, attitudes, and behaviours.
3. How to more effectively engage older women in developing public health responses to preventing and reducing harms from alcohol.
4. Message framings with the greatest potential to encourage changes towards lower risk alcohol consumption.
5. Understandings of policy levers with potential to reduce harms associated with alcohol consumption.

Public health activities should consider the unique needs and potential vulnerabilities of older women drinkers, and how these may be potentially exploited by the tactics of the alcohol industry.

## Data Availability

The datasets generated and/or analysed during the current study are not publicly available as the individuals participating in this survey did not consent for their data to be shared beyond the research team, but are available from the corresponding author on reasonable request.
